# Indoor Air Quality and COVID-19: A Scoping Review

**DOI:** 10.3389/phrs.2023.1605803

**Published:** 2024-01-11

**Authors:** Axelle Braggion, Adeline Dugerdil, Olwen Wilson, Francesca Hovagemyan, Antoine Flahault

**Affiliations:** ^1^ Institut de Santé Globale, Faculté de Médecine, Université de Genève, Geneva, Switzerland; ^2^ School of Public Policy, London School of Economics, London, United Kingdom

**Keywords:** indoor air quality, ventilation, COVID-19, SARS-CoV-2, mitigation strategy

## Abstract

**Objectives:** The COVID-19 pandemic has been a major public health concern for the past 3 years. Scientific evidence on the relationship between SARS-CoV-2 infection and indoor air quality still needs to be demonstrated. This scoping review aims to study the association between air quality indoors and COVID-19.

**Methods:** A scoping review analyzing the association between indoor air quality and epidemiological outcomes was conducted. Papers published between 1 January 2020 and 31 October 2022 were included. Hospital settings were excluded from the study.

**Results:** Eight relevant articles met the inclusion criteria. Indoor settings included workplaces, schools, restaurants, and public transport. Types of ventilation used to improve indoor air quality were dilution methods (opening windows) and mechanical systems with or without filtration or purifier. CO_2_ sensors were employed in one study. All the studies showed a positive association between indoor air quality and its improvement and epidemiological indicators.

**Conclusion:** The findings of this scoping review indicate that indoor air quality, which can be improved with ventilation methods, may reduce the risk of developing COVID-19. Ventilation could thus be viewed as a possible effective mitigating method.

## Introduction

The COVID-19 pandemic has been going on for almost 3 years and the number of confirmed cases has almost reached 648 million worldwide, as of 16 December 2022 [1]. Knowledge about this disease is growing and more than 300,000 scientific publications on the subject can be found on the database *Pubmed*.

Severe acute respiratory syndrome coronavirus 2 (SARS-CoV-2) has several modes of transmission including droplet and airborne [2, 3]. These routes of contagion, particularly aerosols, contribute to the widespread transmission of COVID-19 in indoor spaces. As people from industrialized countries tend to spend most of their time inside [4], indoor transmission has been identified as the main setting for infections [5, 6].

Evidence shows that the spread of other respiratory pathogens, such as influenza and tuberculosis, is contained by adequate air circulation in indoor spaces [7–9]. For example, researchers have demonstrated that after ventilation engineering in a university, carbon dioxide (CO_2_) concentration was greatly reduced and the secondary attack rate for tuberculosis, which is defined as “the number of new cases among contacts” [10], dropped to zero [9]. Another study reported an association between increased incidence of tuberculosis and insufficient ventilation in healthcare facilities [11].

Regarding SARS-CoV-2, much research has investigated air circulation and its role on viral particles [12–16]. Computational fluid dynamics analyses have illustrated that infection risk was reduced when natural or mechanical ventilation techniques were used [12, 13, 15, 16]. Natural techniques include dilution methods, which are commonly defined as supplying fresh air to a room by opening windows and doors [17], whereas mechanical ones include ventilation systems, high-efficiency particulate air (HEPA) filters, and ultraviolet germicidal irradiation (UVGI) [17–20].

Using mathematical models such as the Wells-Riley model, which is an equation describing the airborne transmission of infectious diseases [21], researchers could estimate the risk of airborne contagion in association with indoor air quality [12, 13]. Additionally, several authors have studied the effects of outdoor air quality and SARS-CoV-2 transmission showing that the risk of developing COVID-19 and mortality might be exacerbated by outdoor air pollution [22–26]. Regarding indoor air quality, different recommendations about CO_2_ maximal concentration and the minimum ventilation rate have been published. Indeed, in order to assess proper ventilation, CO_2_ sensors can be installed [27–31]. As they record the level of CO_2_ in parts per million (ppm), they are good proxies of indoor air quality, in addition to being easy to use and inexpensive [29, 30].

The World Health Organization (WHO) recommends a minimum ventilation rate in the context of COVID-19 of 10 L/s/person in non-healthcare and non-residential settings such as workplaces and schools [32]. The American Society of Heating, Refrigerating, and Air-Conditioning Engineers (ASHRAE) recommends a maximum CO_2_ concentration of 700 ppm, whereas the Federation of European Heating, Ventilation and Air Conditioning Associations suggests a higher threshold, with a maximum of 1,000 ppm [33, 34]. However, it is worth noting that CO_2_ level alone is not the only indicator of indoor air quality, and other factors such as humidity, temperature and the presence of other pollutants should also be taken into consideration [35].

Most publications studying indoor air quality and SARS-CoV-2 transmission are simulations and are thus not based on field investigations, meaning the association between improved indoor air quality, e.g., with ventilation and epidemiological indicators such as COVID-19 incidence has yet to be addressed. As hospitals are exceptional settings due to their ventilation requirements and nosocomial infections [36–40], this scoping review only addresses studies conducted indoors, elsewhere than hospitals.

The purpose of this scoping review is to study the association between air quality, which can be improved with ventilation, and COVID-19 in different types of indoor environments.

## Methods

### Search Strategy

**FIGURE 1:**
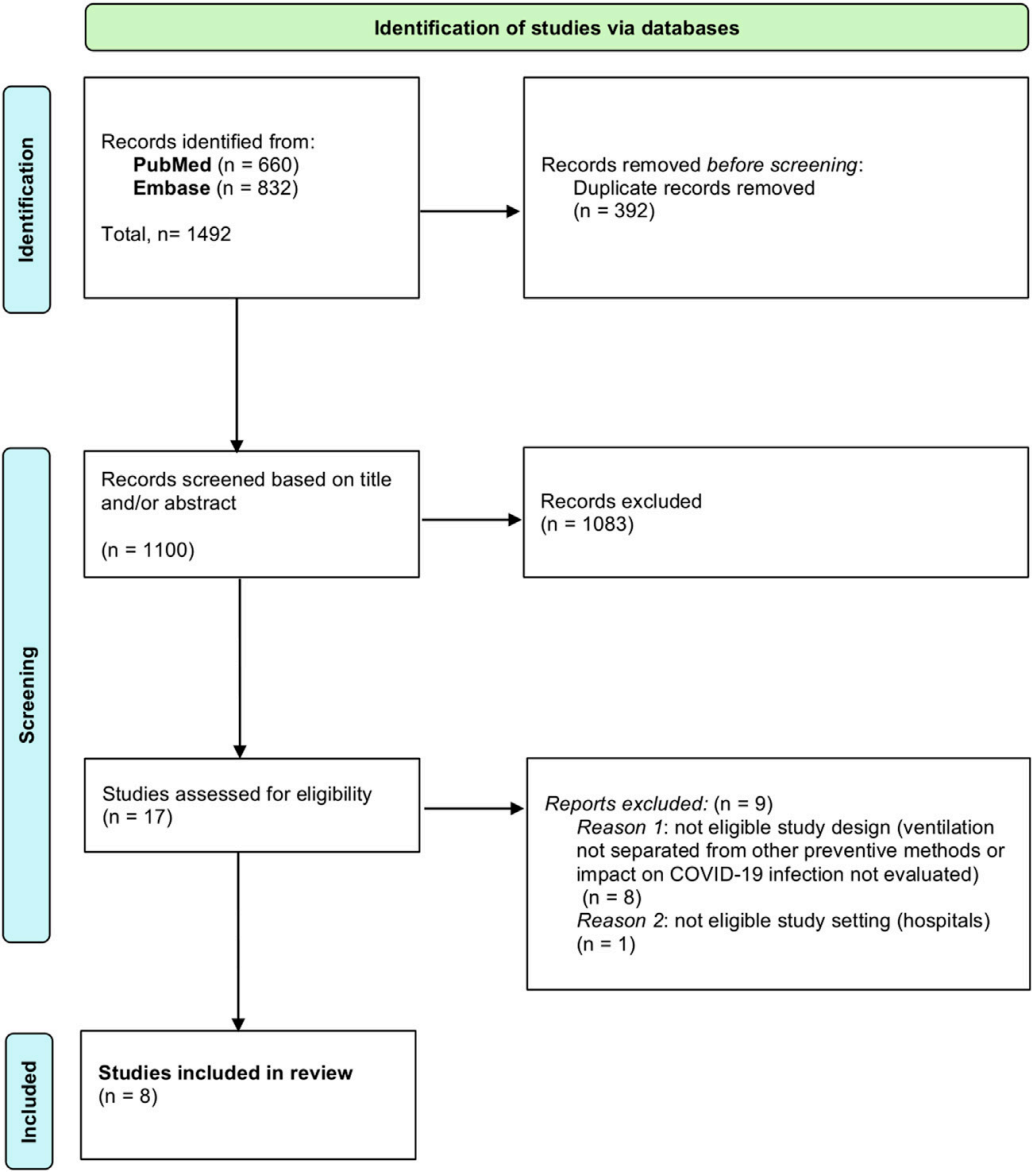
PRISMA flowchart (Scoping review. Global, 1 January 2020 and 31 October 2022).

A scoping review of the literature was conducted to analyze the association between indoor air quality and epidemiological outcomes regarding SARS-CoV-2, following the guidelines from the Preferred Reporting Items for Systematic Review and Meta-analysis for Scoping Reviews (PRISMA-ScR) [41]. Relevant publications were searched for through the *Pubmed* and *Embase* databases. The scoping review, which was conducted on 23 November 2022, was centered on one search round on each database and is outlined in Figure 1 (PRISMA chart). Research queries used mainly contained the same terms but were adapted to the characteristics of each database. They are both described in Appendix 1 (Research queries).

After screening all the identified articles through titles and/or abstracts, the second round was narrowed to search for relevant publications through full-text reading.

### Inclusion and Exclusion Criteria

The included publications had to be written in English or French and published within the study period of 1 January 2020 and 31 October 2022. The scientific papers had to be original research articles either interventional or observational studies, as well as reviews and guidelines. Their primary aim had to address the association between indoor air quality and its improvement through ventilation and SARS-CoV-2 epidemiological outcomes including the number of primary and secondary cases, the number of hospitalizations and the number of deaths related to COVID-19. The studies had to be related to any indoor spaces except hospitals, such as: schools, offices, restaurants and public transport. Furthermore, the following exclusion criteria were applied during the screening: studies constructed as simulations and not based on real infections, papers on indoor air quality monitoring which do not address airborne transmission, and publications studying SARS-CoV-2 transmission through ventilation systems such as in intensive care units. Indeed, these highly medicalized places are too unique and the findings in such special settings cannot be generalized to be included in our scoping review.

### Data Extraction

The identified dataset was extracted and duplicates were removed using *EndNote20* software. The remaining publications were imported into the *Rayyan* web-tool where the screening based on titles and/or abstracts was completed by the main author. In the second stage, the full text of selected articles was read and relevant data were extracted into an *Excel* program (*2011*, *version 14.5.2*). Extracted information included author(s) names, date of publication, study design, study location, date of SARS-CoV-2 outbreak, study period of investigation, type of indoor setting, type of ventilation used, sample size, epidemiological outcome studied, and main results of the study. A second researcher carried out a second reading of the selected articles and a verification of the extracted information.

## Results

As described in the PRISMA flowchart (Figure 1), the last research carried out on 23 November 2022 identified a total of 1,492 records (660 from *Pubmed* and 832 from *Embase*), among which 392 duplicates were removed. After screening based firstly on title and/or abstract and then by full-text reading, 1,484 publications were excluded. Finally, eight relevant articles met the inclusion criteria and were included in the scoping review.

Half of the included papers were published in 2021 [42–45] and the other half in 2022 [46–49]. Regarding the study location, 5 out of 8 were performed in Asia [44, 45, 47–49], 2 in Europe [43, 46] and 1 in the United States [42].

Six studies had an observational design [42, 43, 45–47, 49] and two were interventional [44, 48]. Observational setting included parameters of the ventilation system collected on site [43, 47, 49], self-reported practicing room ventilation assessed by always/often/rarely/never [45] agent-based epidemiological model with/without ventilation [46] or implementing ventilation strategies [42]. One of the interventional study used experimental measures of air conditioner, electric fans, doors closed and CO_2_ sensors to reproduce the ventilation frequency at the time of the cluster [48]. The other one used experimental tracer gas measurement along with computer simulations under various combinations of windows open or closed and air conditioning or heating [44].

Researchers analyzed different epidemiological outcomes. Positive polymerase chain reaction (PCR) tests were used in three studies [46, 48, 49]. Two papers indicated their outcome as self-reported positive test which was a PCR or rapid antigen one [42] or a not specified test [43]. Secondary cases of SARS-CoV-2 infection were studied by two articles, which identified these secondary cases, using contact tracing [44, 47] and phylogenetic analysis [47]. Self-reported COVID-19 like symptoms, which included cough, sore throat, dyspnea, smell/taste changes or fever (>37.5°C) were studied by one article [45]. None of the included articles studied hospitalization or mortality rate.

Several types of indoor settings were investigated including workplaces [43, 45, 48, 49], schools [42, 46], restaurants [47] and public transport [44].

Different kinds of ventilation were used such as opening windows and doors [45], ultraviolet germicidal air purification (UVGI) [47] and unspecified type of ventilation [46]. Some researchers studied a combination of ventilation methods, for example: dilution method (opening doors, opening windows, using fans to increase effectiveness of open windows) and/or the installation of high-efficiency particulate air (HEPA) filtration systems and/or the installation of UVGI [42]. Another study looked at conditioning/heating associated with opening windows [44] and two analysis included an unspecified type of ventilation equipment along with open windows and doors [48, 49]. Only one of the selected publications used CO_2_ sensors in their study [48].

No guidelines nor reviews were included as none was fulfilling the purpose of our scoping review.

All of the selected studies showed an association between ventilation and their investigated epidemiological outcome. The main extracted data is displayed in Table 1.

**TABLE 1:** Description of study design and main results of the included papers, sorted by indoor settings (Scoping review. Global, 1 January 2020 and 31 October 2022).

	Type of specific indoor setting	Study design	Type of ventilation method	Type of epidemiological outcome	Sample size	Main findings
Workplaces						
Pokora R. et al. 06.2021	Meat and poultry processing plants	Observational: processing plants with vs. without ventilation	Mechanical: ventilation system	Self-reported positive test	19,072 employees, including 880 tested positive	Having a ventilation system reduced the chance of testing positive (aOR of 0.757, 95% CI 0.563–1.018)
						Adjusted with comply with minimum distance of 1.5 m, room temperature, type of work break (shifted or not) and type of contractual relationship (regular or temporary)
Shimizu S. et al. 12.2021	Non health related workplaces settings	Observational: rarely/never vs. always practising ventilation	Dilution: opening windows and doors	Self-reported COVID-19-like symptoms (cough, sore throat, dyspnea, smell/taste changes, fever (>37.5°C)	19,941 included participants	Rarely/never practicing room ventilation was significantly associated with all COVID-19-like symptoms, except fever (aOR of 1.38, 95% CI 1.26–1.52)
						Adjusted with age, sex, educational attainment, annual household income, marital status, job type, smoking status, underlying diseases, and regional incidence rate of COVID-19
Kitamura H. et al. 04.2022	Manufacturing factory	Interventional: experimental measures of ventilation and CO_2_ sensors	Dilution: opening windows and doors, electric fans	Positive PCR test	5 tested positive cases	The estimated air change per hour (ACH) was 0.74 under the conditions of the emerged cluster, which is less than the 2 ACH recommended by the local ministry of Health. This suggests an increased risk of airborne transmission
			Mechanical: ventilation equipment			
Cho S. H. et al. 08.2022	Ship (amphibious warfare from the Republic of Korea Navy)	Observational: floor with vs. without ventilation	Dilution: unspecified natural ventilation on 2nd and 3rd floor Mechanical: ventilation system	Positive PCR test	85 crew members, including 38 tested positive	Risk of SARS-CoV-2 infection was increased when comparing basement and 1st floor (without any sort of ventilation) with 2nd and 3rd floor (aOR 3.20, 95% CI 1.14–8.99) Adjusted with age, rank (in the Navy) and sex
Schools						
Gettings J. et al. 05.2021	Schools	Observational: schools with vs. without ventilation strategies	Dilution: opening doors, opening windows, using fans to increase effectiveness of open windows	Self-reported PCR or rapid antigen positive test	91,893 students in 169 schools	COVID-19 incidence was lower with improved ventilation (RR 0.61, 95% CI 0.43–0.87, when adjusting for county-level incidence)
			Mechanical: HEPA filtration systems, UVGI			
Lasser J. et al. 01.2022	Schools	Observational: agent-based epidemiological model with vs. without ventilation	Unspecified room ventilation	Positive PCR test	9,232 cases tested positive	Room ventilation allowed the largest reduction in cluster size, compared to other preventive measures (estimated 64% reduction in transmission risk)
Restaurants						
Cheng V.C. et al. 02.2022	Restaurants	Observational: restaurant with vs. without ventilation	Mechanical: Ultraviolet-C air purifiers	Secondary cases of COVID-19 identified by contact tracing, phylogenetic analysis and PCR testing	2 restaurants including 283 clients and 41 staff members	Lower secondary attack rate among customers in restaurant with air purifiers (3.4% versus 28.9%)
Public transport						
Ou C. et al. 10.2021	Buses	Interventional: experimental tracer gas measurements with vs. without ventilation	Dilution: opening windows	Secondary cases of COVID-19 identified by contact tracing and PCR testing	2 buses including 62 passengers	Lower secondary attack rate on the more ventilated bus (11.8% versus 15.2%)
			Mechanical: air conditioning/heating			

OR, adjusted odds ratio; RR, rate ratio; 95% CI, confidence interval; PCR, polymerase chain reaction; HEPA, high-efficiency particulate air; UVGI, ultraviolet germicidal irradiation.

## Discussion

This scoping review was conducted to study the association between improved air quality through ventilation methods and COVID-19 in indoor settings. As highlighted by the results of all the included studies, indoor air quality and its improvement with ventilation was associated with better COVID-19 epidemiological outcomes.

Despite the thousands of articles that have been published on the transmission of SARS-CoV-2, few have specifically focused on evaluating the association between indoor air quality and developing COVID-19. Indeed, out of 1,100 publications identified through our search strategy, only eight papers addressed this specific topic. This lack of research might be explained by the fact that scientific and political resources have been focused on the development of effective vaccines and promoting mask use.

Schools are known to be hotspots for the spread of infectious diseases, especially airborne pathogens [15, 50]. Poor ventilation and low indoor air quality are thought to be the driving forces of viral transmission in educational buildings. In order to decrease CO_2_ levels in classrooms, recommendations regarding ventilation have been made by different organizations, including the World Health Organization (WHO) and the Centers for Disease Control and Prevention (CDC) [51, 52]. In our included articles, a report from the State of Georgia (United States) found a reduction in COVID-19 incidence for schools that reported using dilution methods [42]. However, their analysis did not specify whether face masks were also required in schools that improved ventilation, which could be a confounding factor. Nevertheless, another study conducted in Austrian schools isolated room ventilation from other mitigating strategies and noted a reduction in cluster size in transmission risk [46]. These two articles included in our review, did not employ CO_2_ sensors as proxies of air quality in classrooms. Indeed, CO_2_ monitors can help ensure proper ventilation inside educational spaces [53, 54]. Schools could thus aim to improve air quality in classrooms with ventilation methods in order to decrease the risk of exposure to SARS-CoV-2 contaminated particles. Educational places could use CO_2_ sensors as indirect tools of proper ventilation, In workplaces, poor ventilation is also associated with more cases of SARS-CoV-2 infections across different work settings [43, 45, 48, 49]. A Japanese study found a positive association between the occurrence of any COVID-19 like symptoms among workers who reported they never or rarely implemented room ventilation [45]. However, the symptoms were self-reported and therefore could be due to other respiratory viruses. Polymerase Chain Reaction (PCR) tests would have been required to ensure that these symptoms were the result of SARS-CoV-2 infection.

Another study carried out in a manufacturing plant has demonstrated that at the time of a cluster, the ventilation inside the building was less than the local recommendations [48]. This is surprisingly the only included paper in our scoping review that used CO_2_ sensors in their analysis. These monitors revealed that adequate ventilation significantly decreased CO_2_ concentration. These findings support the possible association between poor ventilation, polluted air and SARS-CoV-2 contamination. As proxies of indoor air quality, CO_2_ monitors could be used more systematically in enclosed and crowded settings. Indeed, they could help ensure proper ventilation and thus reduce the risk of SARS-CoV-2 airborne transmission.

Further evidence to reinforce this relationship comes from a warfare ship from the Republic of Korea’s Navy [49]. They investigated a COVID-19 outbreak and found that the floors without ventilation had a significantly greater risk of group infection. Similarly, research into transmission at meat and poultry plants found that the chance of testing positive for COVID-19 was reduced when using a ventilation system [43]. As a computational fluid dynamics simulation demonstrated, workplace design and open offices in particular should be adapted in order to promote natural ventilation through opening windows and to decrease infection risk for workers [54, 55].

In restaurants and bars, transmission may be enhanced because mitigation strategies such as wearing a face mask and keeping a safe distance are not possible [47, 56, 57]. A study conducted in Hong Kong highlighted that using ultraviolet-C air purifiers decreased the secondary attack rate among customers [47]. Nevertheless, there were some confounding factors such as the different architecture of the two restaurants. Furthermore, their customers differed as none of the secondary cases were vaccinated in one of the restaurant whereas they all had two doses of a COVID-19 vaccine in the second one. However, vaccination is acknowledged to be poorly effective against mild infections and transmission, particularly against the Omicron variant (B.1.1.529) [58]. The variant of SARS-CoV-2 involved in the two restaurants’ outbreak differed. Despite the Omicron (B.1.1.529) variant’s higher transmissibility, the air purifiers may have contributed to reducing the secondary attack rate, compared to lower-transmissibility variant (Delta variant B.1.617.2) and no air purifiers. These findings suggest that, regardless of vaccination rate and SARS-CoV-2 variant, adequate air purification could effectively reduce the risk of COVID-19 infection in restaurants. As face masks cannot be used while eating [56], restaurants managers could work with CO_2_ sensors as proxies of proper ventilation.

Finally, public transport is the last indoor space evaluated in our review. These enclosed spaces are known to be at high risk of respiratory virus transmission due to overcrowding, extended duration of exposure to contaminated individuals, recirculation of infected air, and poor ventilation [59, 60]. This problematic setting has been recognized by the WHO, which, in November 2020, published recommendations for governments, transport providers, and commuters on minimizing transmission on public transport [61]. One study from China examined a COVID-19 cluster and showed that a bus with a lower ventilation rate had a higher secondary attack rate [44]. Since the index case was the same, these findings suggest that the poorer ventilation could explain the greater number of secondary infections, although exposure time could be a confounding factor. On the other hand, fewer individuals were wearing face masks in the bus with the higher secondary attack rate, which strengthens the possible association between ventilation and reduced risk of developing COVID-19. Experimental studies from trains confirm that adequate ventilation decreased the propagation of airborne particles and thus reduce risks of SARS-CoV-2 transmission [62, 63].

This study is the first, to our knowledge, to review the association between indoor air quality and COVID-19 epidemiological outcomes. Our literature screening included articles published during the three first years of the pandemic (2020–2022) and was not restricted to a specific indoor environment such as schools or hospitals. A limitation of this review is the use of only two databases (*Pubmed* and *Embase*) and one reviewer to screen the publications, which may miss out other studies. Additionally, the research exclusively focused on articles written in English or in French and thus relevant publications published in other languages might have not been included. Weaknesses related to the study design (scoping review) such as non-exhaustive search and broad results and no definitive causal conclusions should also be mentioned. Furthermore, this study design does not allow definitive causal conclusions. Lastly, we excluded studies conducted in hospitals because of the special setting of these spaces (ventilation systems and nosocomial infections [36–40]) which was intended although it could be noted as another limitation.

As we could only find a few papers on the subject and as the design of our study does not assert a definitive cause-and-effect relationship between SARS-CoV-2 infection and indoor air quality, further analysis should be conducted. Furthermore, since randomized controlled trials are uncommon in such domains compared to, e.g. drug development, further studies from different research groups and from various parts of the world should help draw evidence on the causal relationship between indoor air quality and the risk of airborne pathogens infection such as SARS-CoV-2.

### Conclusion

The COVID-19 pandemic has highlighted the importance of airborne transmission. Improving indoor air quality with ventilation methods is associated with a reduced risk of SARS-CoV-2 infections. Further analyses are needed to strengthen the evidence related to the relationship between poor air quality indoors and developing COVID-19.
