# Supplementary material for: Indoor Air Quality and COVID-19: A Scoping Review
Source: Public Health Rev. 2024 Jan 11;44:1605803. doi: 10.3389/phrs.2023.1605803 (PMC10810127; doi:10.3389/phrs.2023.1605803)
Supplement: Supplementary file 1 [file DataSheet1.pdf]

Appendix 1: Research queries

- <u>Research query used on *Pubmed*</u>:

("SARS-CoV-2"[Mesh] OR "COVID-19"[Mesh] OR "SARS-CoV-2"[tiab] OR covid[tiab] OR "2019 Novel Coronavirus Infection"[tiab] OR "2019-nCoV"[tiab] OR "Coronavirus disease 2019"[tiab] OR "Severe Acute Respiratory Syndrome Coronavirus 2"[tiab] OR "SARS Coronavirus 2"[tiab]) AND ("Air Pollution, Indoor"[Mesh] OR (("Ventilation"[Mesh] OR "Environment, Controlled"[Mesh:NoExp] OR "Carbon Dioxide/analysis"[Mesh] OR "Air Conditioning"[Mesh] OR "CO2 monitor*"[tiab] OR "CO2 sensor*"[tiab]) AND ("Built Environment"[Mesh] OR "Restaurants"[Mesh] OR "Schools"[Mesh:NoExp] OR "Universities"[Mesh] OR "Sports and Recreational Facilities"[Mesh] OR "Workplace"[Mesh] OR "Transportation Facilities"[Mesh] OR indoor*[tiab] OR "Built Environment*"[tiab] OR buildings[tiab] OR restaurant*[tiab] OR classroom*[tiab] OR school*[tiab] OR campus[tiab] OR "Fitness Center*"[tiab] OR "sport center*"[tiab] OR "sport facilit*"[tiab] OR "Swimming Pool*"[tiab] OR workplace*[tiab] OR office*[tiab] OR train[tiab] OR trains[tiab] OR bus[tiab] OR buses[tiab] OR "public transport*"[tiab])) OR "indoor air quality"[tiab] OR "IAQ"[tiab] OR "indoor air monitoring"[tiab] OR "indoor environmental quality"[tiab] OR "room air quality"[tiab] OR "indoor environmental control"[tiab] OR "natural ventilation"[tiab] OR "air circulation"[tiab] OR "air recirculation"[tiab] OR "air circulation"[tiab] OR "adequate ventilation"[tiab] OR "air filtration"[tiab] OR "room ventilation"[tiab] OR "indoor ventilation"[tiab] OR (ventilat*[ti] AND ("Restaurants"[Mesh] OR "Schools"[Mesh:NoExp] OR "Universities"[Mesh] OR "Sports and Recreational Facilities"[Mesh] OR "Workplace"[Mesh] OR "Transportation Facilities"[Mesh] OR indoor*[tiab] OR buildings[tiab] OR restaurant*[tiab] OR classroom*[tiab] OR school*[tiab] OR campus[tiab] OR "Fitness Center*"[tiab] OR "sport center*"[tiab] OR "sport facilit*"[tiab] OR "Swimming Pool*"[tiab] OR workplace*[tiab] OR train[tiab] OR trains[tiab] OR bus[tiab] OR buses[tiab] OR "public transport*"[tiab]))) AND (English[la] OR French[la])

- <u>Research query used on *Embase*</u>:

('coronavirus disease 2019'/exp OR 'severe acute respiratory syndrome coronavirus 2'/exp OR ("SARS-CoV-2" OR covid OR "2019 Novel Coronavirus Infection" OR "2019-nCoV" OR "Coronavirus disease 2019" OR "Severe Acute Respiratory Syndrome Coronavirus 2" OR "SARS Coronavirus 2"):ab,ti,kw) AND ('indoor air quality'/de OR 'indoor air pollution'/de OR 'room ventilation'/de OR 'aeration'/de OR 'workroom air'/de OR 'air cleaning system'/de OR (('air analysis'/exp OR 'air monitoring'/de OR 'air quality control'/de OR 'air pollution control'/de OR 'carbon dioxide gas analyzer'/exp OR 'air conditioning'/de OR (CO2 NEAR/3 (monitor* OR sensor* OR indoor*)):ab,ti,kw) AND ('indoor environment'/de OR 'built environment'/de OR 'restaurant'/de OR 'school'/exp OR 'sport facility'/exp OR 'workplace'/de OR (indoor* OR "Built Environment*" OR buildings OR restaurant* OR classroom* OR school* OR campus OR "Fitness Center*" OR "sport center*" OR "sport facilit*" OR "Swimming Pool*" OR workplace* OR office* OR train OR trains OR bus OR buses OR "public transport*"):ab,ti,kw)) OR (room* NEAR/3 air NEAR/3 (quality OR monitor* OR circulation OR recirculation OR filtration)):ab,ti,kw OR ((air NEAR/3 (quality OR monitor* OR circulation OR recirculation OR filtration)):ab,ti,kw AND ('indoor environment'/de OR 'built environment'/de OR 'restaurant'/de OR 'school'/exp OR 'sport facility'/exp OR 'workplace'/de OR (indoor* OR "Built Environment*" OR buildings OR restaurant* OR classroom* OR school* OR campus OR "Fitness Center*" OR "sport center*" OR "sport facilit*" OR "Swimming Pool*" OR workplace* OR office* OR train

OR trains OR bus OR buses OR "public transport*"):ab,ti,kw)) OR (ventilation NEAR/6 (natural OR adequate OR room* OR indoor* OR "Built Environment*" OR buildings OR restaurant* OR classroom* OR school* OR campus OR "Fitness Center*" OR "sport center*" OR "sport facilit*" OR "Swimming Pool*" OR workplace* OR office* OR train OR trains OR bus OR buses OR "public transport*")):ab,ti,kw) NOT ('conference abstract'/it OR 'conference review'/it) AND ([english]/lim OR [french]/lim)
